# ECG Screening in Athletes: A Systematic Review of Sport, Age, and Gender Variations

**DOI:** 10.31083/RCM38209

**Published:** 2025-05-28

**Authors:** Adela Caramoci, Alina Maria Smaranda, Teodora Simina Drăgoiu, Ioana Anca Bădărău

**Affiliations:** ^1^Discipline of Sports Medicine, Carol Davila University of Medicine and Pharmacy, 050474 Bucharest, Romania; ^2^National Institute of Sports Medicine, 022103 Bucharest, Romania; ^3^Faculty of Medicine, Carol Davila University of Medicine and Pharmacy, 050474 Bucharest, Romania; ^4^Department of Physiology, Carol Davila University of Medicine and Pharmacy, 050474 Bucharest, Romania

**Keywords:** electrocardiography, athletes, sports cardiology, ECG screening

## Abstract

**Background:**

The electrocardiogram (ECG) screening in athletes is essential due to the unique cardiac adaptations induced by intensive training. However, differentiating between physiological adaptations and pathological abnormalities remains a significant challenge, particularly when considering variations across different sports, ages, and genders.

**Methods:**

A systematic review of observational studies published between 2015 and 2025 was conducted according to the Preferred Reporting Items for Systematic Reviews and Meta-Analyses (PRISMA) guidelines. Data were extracted from 20 studies examining ECG changes in athletes across endurance, strength, and mixed sports, encompassing both adolescent and adult populations.

**Results:**

Commonly observed ECG changes included increased QRS amplitude, T-wave inversions, and sinus bradycardia, particularly in endurance athletes, while strength-based athletes frequently exhibited left ventricular hypertrophy. Male athletes showed higher QRS voltages, longer QRS durations, and higher PR intervals, whereas female athletes demonstrated elevated resting heart rates and prolonged corrected QT interval (QTc) intervals. Adolescents who engaged in regular sports displayed fewer abnormal ECG findings than adults; however, high-intensity training in adolescent athletes was associated with right atrial enlargement and increased P-wave duration. Detraining effectively reversed certain ECG changes, including prolonged QT intervals and T-wave abnormalities, though these changes often reappeared upon resumption of intense training. Notably, *de* *novo* ECG abnormalities, such as T-wave inversions and ST-segment depression, were identified in athletes with post-COVID-19 infections. This review also highlights the financial burden of widespread ECG screening, but reinforces the importance of ECG screening in preventing sudden cardiac death (SCD) through comprehensive cardiac evaluations.

**Conclusion:**

This review emphasizes the complexity of ECG interpretation in athletes, highlighting sport-specific, gender-based, and age-related variations. The persistent high false-positive rates underscore the need for refined, sport-specific ECG guidelines. Recent recognition of sports medicine as a primary specialty within the European Union (EU) reinforces the importance of comprehensive physician training. Integrating artificial intelligence (AI) technology into ECG screening can enhance diagnostic accuracy, reduce costs, and facilitate large-scale implementation. Meanwhile, collaborative efforts among clinicians, researchers, and policymakers are essential to developing cost-effective and standardized ECG screening protocols, ensuring improved athlete care, and advancing the field of sports cardiology.

## 1. Introduction 

The electrocardiogram (ECG) is an indispensable tool in cardiology, serving as a cornerstone in the diagnosis and management of cardiac pathologies, including myocardial infarction, congestive heart failure, and arrhythmias [1]. The ECG’s utility extends beyond pathological diagnosis; it is also employed in evaluating healthy individuals during fitness assessments, pre-employment screenings, and before participation in sports events. Pre-participation medical evaluation (PPE) for elite athletes is crucial, as highlighted in recent research [2], which emphasizes the role PPE in ensuring safe participation in sports, detecting potential complications early, and facilitating personalized medical monitoring. This underscores the significance of ECG screening in the context of sports medicine, aligning with European standards for athlete health assessments. Interpreting ECG among athletes presents unique challenges due to physiological adaptations resulting from intensive training [3, 4]. Sports and Exercise Medicine (SEM) is a multidisciplinary specialty supporting athlete performance while ensuring health through regular PPE and medical monitoring, as highlighted by the European Federation of Sports Medicine Associations (EFSMA) through the Scientific and Educational Commission. ECG screening is a critical component of PPE, especially in Europe where it is strongly recommended, despite debates about its necessity in all athletes [2]. SEM professionals aim to detect life-threatening complications, optimize performance, and guide training and recovery strategies, further reinforcing the significance of ECG in SEM. Advances in digital ECG interpretation have enhanced accuracy, making it more reliable than traditional visual analysis alone [5].

Athletes frequently exhibit specific ECG alterations, including increased QRS voltage, early repolarization patterns, and sinus bradycardia. These findings are attributed to cardiac anatomical and physiological changes, such as cardiac enlargement and heightened vagal tone mediated by the vagus nerve [6]. In moderate cases, these adaptations may result in left ventricular hypertrophy (LVH), evident through increased QRS amplitude and T-wave inversions on the ECG [7, 8]. Severe cases may progress to hypertrophic cardiomyopathy (HCM), a condition associated with significant clinical complications [9].

Differentiating between physiological adaptations and pathological abnormalities is critical in clinical practice. Physicians must balance avoiding unnecessary interventions with detecting insidious cardiac pathologies that may predispose athletes to sudden cardiac death (SCD) [10]. The International Criteria for Electrocardiographic Interpretation in Athletes, established by Drezner *et al*. [11], provides updated consensus standards to aid physicians in recognizing normal athletic adaptations versus pathological findings, enhancing the accuracy of athlete ECG assessments, and supporting sport-specific cardiac evaluations. Additionally, Ragazzoni *et al*. [12] highlighted the need for specific ECG interpretation guidelines in pediatric athletes, proposing an algorithm that helps differentiate normal, borderline, and abnormal findings, thereby enhancing early diagnosis and management of potential cardiac conditions.

This review aimed to explore recent advances and address new questions in the field: What novel insights have emerged in the past decade regarding ECG changes in athletes across different sports? How do contemporary studies differentiate ECG patterns in adolescents versus adults? Has recent research clarified the influence of sports categories (endurance, strength, mixed) on ECG outcomes? New findings have also highlighted emerging factors such as gender-based variations, training intensity effects, and post-coronavirus disease 2019 (COVID-19) cardiac changes, reflecting the evolving understanding of athletic cardiac adaptations in recent years.

The objectives of this review are to critically analyze recent literature regarding ECG findings in athletes. It emphasizes the importance of periodic reviews to refine and enhance interpretation guidelines. By synthesizing contemporary data, this review aims to highlight the evolving challenges and advancements in distinguishing physiological adaptations from pathological findings, thereby supporting the development of accurate and sport-specific ECG interpretation standards.

## 2. Literature Review

### 2.1 Methodology

This systematic review employed the Preferred Reporting Items for Systematic Reviews and Meta-Analyses (PRISMA) guidelines to ensure a transparent and replicable methodology. The objective was to collect and evaluate peer-reviewed studies published between 2015 and 2025 that investigated ECG changes in athletes across various sports. Inclusion criteria encompassed observational studies focusing on endurance, strength, and mixed sports in adolescent and adult athletes. Studies not reporting ECG findings, involving athletes with cardiac comorbidities, or classified as editorials, commentaries, or review papers were excluded.

PubMed/Medline was used as the primary electronic database for article retrieval. Other databases included Cochrane Reviews, PEDro, and the Google Scholar search engine. The search itself involved the use of a combination of Medical Subject Headings (MeSH) terms and/or keywords in the Title and abstracts, that were related to ECG, athletes, and sport types. The search strategy is presented in Table 1 while the PubMed search outcomes can be seen in Appendix Table 4.

**Table 1.** **Search strategy keywords**.

Search #	Search term
#1	electrocardiography [MeSH Terms]
#2	athletes [MeSH Terms]
#3	sports [MeSH Terms]
#4	endurance sports [Title/Abstract]
#5	strength sports [Title/Abstract]
#6	mixed sports [Title/Abstract]
#7	ECG changes [Title/Abstract]
#8	screening [Title/Abstract]
#9	#1 AND #2
#10	#1 AND #3
#11	#1 AND #4 OR #5 OR #6
#12	#1 AND #2 AND #7
#13	#1 AND #2 AND #8

MeSH, Medical Subject Headings; ECG, electrocardiogram.

Duplicates were removed using Zotero. Two reviewers independently screened the titles and abstracts, with a third reviewer consulted to resolve any discrepancies. Data extraction included information on athlete demographics, sport type, training level, and ECG findings, organized in Microsoft Excel. The quality of the study was assessed using the Newcastle-Ottawa Scale, without consideration of a bias risk. Since the data was publicly available, ethical approval was not required.

### 2.2 Results

**Fig. 1.**
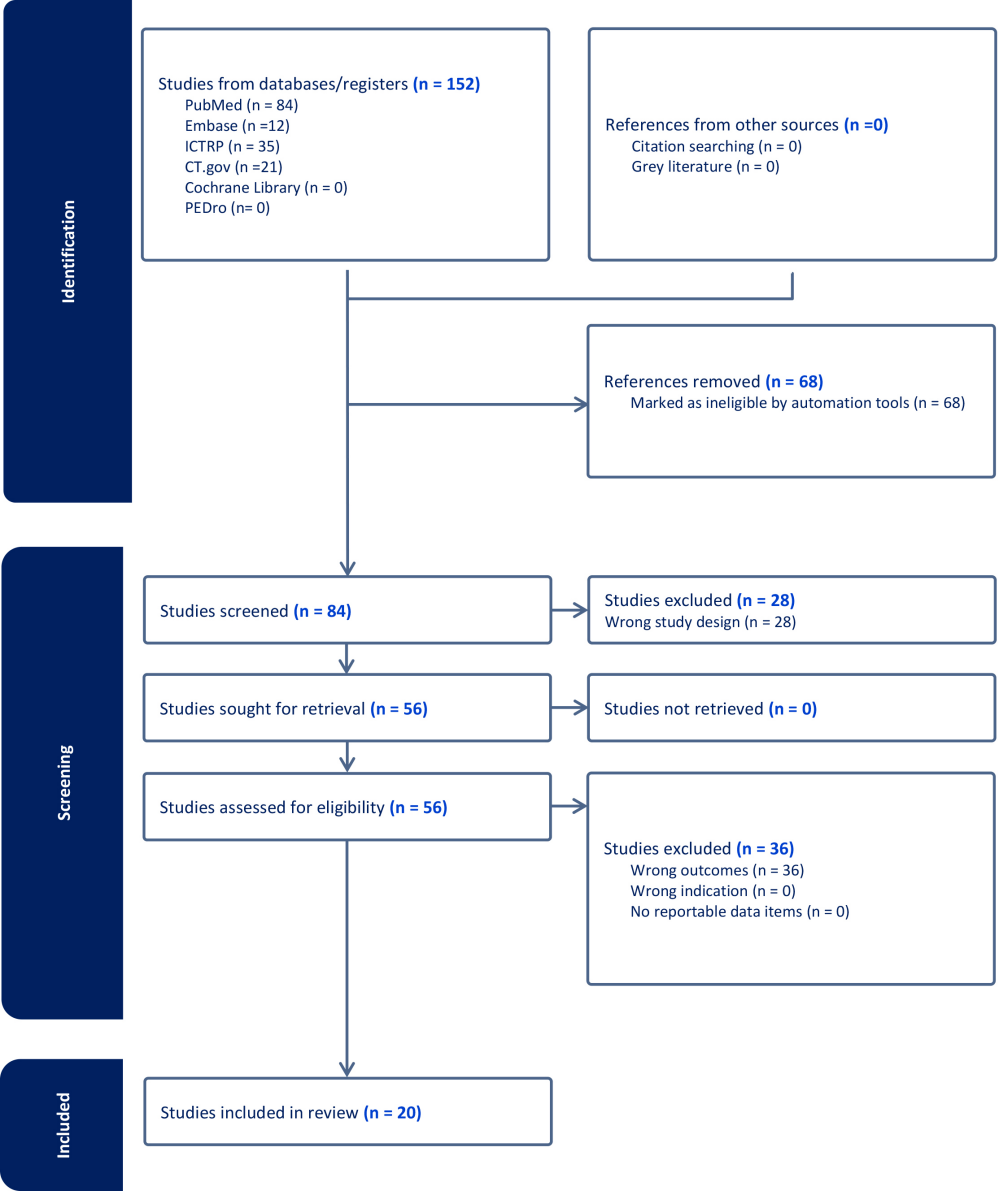
**PRISMA flowchart**. PRISMA, Preferred Reporting Items for Systematic Reviews and Meta-Analyses; PubMed, Public/Publisher MEDLINE; Embase, Excerpta Medica Database; ICTRP, International Clinical Trials Registry Platform; CT.gov, ClinicalTrials.gov; PEDro, Physiotherapy Evidence Database.

From all 20 studies [13, 14, 15, 16, 17, 18, 19, 20, 21, 22, 23, 24, 25, 26, 27, 28, 29, 30, 31, 32] found, we noticed recurrent themes (Fig. 1). These included common ECG changes along with their reversibility, consistent gender, and age differences in ECG findings, sports-specific adaptations, as well as risk factors.

The common ECG changes noted included QRS amplitude and T-wave inversions (TWI) which were often associated with left ventricular hypertrophy. Sinus bradycardia was consistently reported in most athletes. In endurance athletes, early repolarization patterns were frequently observed. Most interestingly certain studies reported prolongation of QT interval, but this was reversed following ‘de-training’ (Table 2, Ref. [13, 14, 15, 16, 17, 18, 19, 20, 21, 22, 23, 24, 25, 26, 27, 28, 29, 30, 31, 32]).

**Table 2.** **Data synthesis from observational studies**.

Study	Study type	Country	Sample Size	Age range	Male number	Sport type	Training level	Main findings	Duration of follow-up	Limitations
[13, 14]	Retrospective observational study	Switzerland	891 pediatric athletes	Mean age 14.8 years (range 8 to 17)	65% male	Various sports, predominantly football	Youth athletes	Abnormal findings including T-wave inversion	6 years	Limited to Swiss pediatric athletes; potential selection bias; findings may not be generalizable to other populations. The cost of screening was 122 USD per athlete.
								19 athletes (2.1%) had abnormal ECG findings, predominantly males, performing endurance sports; further investigations found no relevant pathology.		
[15]	Observational cross-sectional	Netherlands	1436	18–19	72% males	College sports	Medical clearance before joining a college sports program	Higher PR intervals, lead voltages, and QRS duration in males. Higher resting heart rates, and QTc intervals in females.	Not specified	Only included young athletes from a specific program.
								Gender-related ECG differences in young athletes.		
[16]	Observational study	UK	511 soccer players	Median age 21 years (18–26 years)	Not specified	Soccer	Elite athletes	*De-novo* ECG changes including reduction in T-wave amplitude, T-wave inversion, and ST-segment depression	Not specified	Limited to elite soccer players; potential selection bias; findings may not be generalizable to other sports or athlete populations; follow-up duration not specified.
								3% of athletes demonstrated *de-novo* ECG changes post-COVID-19 infection; 88% were diagnosed with cardiac inflammation		
[17]	Observational retrospective	Italy	310	Median age 14 (range 6–25)	65% males	Various sports	Intensive weekly training	Prolonged QT interval, T- wave abnormalities	3–4 months, up to 7 months for some	Limited to those actively practicing sports, excludes long-term follow-up.
								Many QTc normalized after detraining; 40% reverted with retraining. Considered acquired LQTS.		
[18]	Observational cohort	Spain	6401	Mean ± SD age 11.2 ± 2.9 years (range 5–16 years)	93.8% males	Soccer	Pre-participation screening (PPS)	CSP: QRS ≤100 ms, S wave <40 ms in I or V6 together with an RSR pattern in lead-V1	Not specified	Limited to children attending a PPS program within a specific period.
								A higher proportion of athletes with CSP compared to IRBBB. CSP might have been misdiagnosed as IRBBB.		
[19]	Observational Study	USA	1596	16.2–26.3	708	High school, college, and professional athletes	Mixed	High rate of positive responses to AHA guidelines, varying ECG results based on interpretation criteria.	Not specified	High false positive rate, regional limitation, need for updated screening guidelines.
[20]	Cohort Study	Norway	81	35–74	63	Endurance sports	High	High negative predictive value of PCVE. Detected CVD in 20% of master athletes, primarily in the symptom group.	5 months	Small sample size, limited number of female participants, self-reported questionnaires.
[21]	Observational study	Spain	356 (308 athletes, 48 controls)	36.4 years (athletes), 49.3 years (controls)	Not specified	Various sports including competitive athletes	Competitive training	Left atrial enlargement is common in adult competitive athletes but not accompanied by a significant modification in electrocardiographic parameters	Not specified	The study is limited to the analyzed population and may not be generalizable to all athletes; potential bias due to sample selection and specific population traits.
								No relevant differences in P-wave duration, prevalence of interatrial block, or morphology-voltage-P-wave duration score		
[22]	Observational Study	France	2457	35+	Not specified	Leisure time sportsmen and sportswomen	Mixed	Positive exercise ECG correlated with higher CVD risk factors, supporting the use of exercise ECG in screening.	3 years	No specific funding for the study, limited to asymptomatic participants.
[23]	Retrospective observational study	Spain	3747 athletes aged 11–16 years	11–16 years	77.5% male	Various sports, predominantly football and basketball	Youth athletes	Low prevalence of abnormal ECGs (2.05%); differences by age and sex; supportive of Seattle criteria for screening young athletes	Not specified	Limited to a specific age group (11–16 years); potential for high false positives; may not be generalizable to other populations or age groups.
[24]	Observational Study	Poland	40 amateur male marathon runners	Not specified	Not specified	Marathon running (amateur)	Amateur runners	Resting ECGs showed abnormalities in 92.5% of subjects, mostly sinus bradycardia; post-exercise ECGs showed right atrial enlargement in 42.5% of subjects	Not specified	Small sample size; limited to amateur male marathon runners; potential selection bias; findings may not be generalizable to other populations.
[25]	Observational Study	USA	2900 college athletes	Mean age 20 years	32% female, 27% black	Various sports	College athletes	Identified major non-COVID-19 cardiovascular pathology in 1/500 athletes; limited value of TTE in athletes with normal ECG	Not specified	Findings limited to college athletes; potential for inconsistent reporting of coronary anatomy and aortic dimensions; may not be generalizable to other populations.
[26]	Comparative study	Poland	67	39 ± 8 (males), 40 ± 7 (females)	40 males, 27 females	Marathon runners	Amateurs undergoing 12-lead electrocardiogram	Increased P-wave amplitude in males post-marathon; QTc prolongation more pronounced in males	Not specified	Small sample size; limited to amateur marathon runners.
[27]	Prospective cohort screening	Canada	1419	Range (12–35 years)	Not specified	Young competitive athletes	SCBC questionnaire and ECG without physical examination	Diagnoses: 4 pre-excitation, 1 long QT syndrome, 1 mitral valve prolapse, 1 hypertrophic cardiomyopathy	Not specified	High number of false positives with physical examination strategy.
[28]	Observational Study	Italy	33 (18 athletes, 15 nonathletes)	23 years (athletes), 32 years (nonathletes)	Not specified	Various sports (mixed and endurance)	Competitive training	Athletes had larger right ventricular unipolar scar areas; lower VA recurrence in detrained athletes compared to nonathletes	18.7 months	Small sample size; limited to a single center; potential selection bias; findings may not be generalizable to other populations; follow-up duration may be insufficient to capture long-term outcomes.
[29]	Observational Study	Finland	154 sports clubs, 100 secondary schools	15.5 years (mean)	Not specified	Various sports	Varies between groups: ‘always athletes’, ‘never athletes’, ‘changers’	Various ECG changes including heart rate, PR interval, QRS duration, QRS axis, QRS amplitude, T axis, QT interval	4 years	The sample size may not be representative of all athletes; potential selection bias; is limited to the Finnish population.
[30]	Observational Study	USA	26 women marathon runners	42–82 years (mean)	Not specified	Marathon running (long-term)	Long-term runners	Long-term marathon running in women is associated with low coronary plaque formation and favorable cardiovascular risk profiles compared to sedentary women	Not specified	Small sample size; limited to women runners; potential selection bias; findings may not be generalizable to other populations or male runners.
[31]	Retrospective Study	Serbia	640	Range (10–14 years)	Not specified	Various (volleyball, soccer, basketball, swimming, etc.)	Mixed	Increased number of ECG findings requiring additional cardiac examination in young athletes during COVID-19.	Not specified	Limited to young athletes, the potential influence of COVID-19 on results.
[32]	Observational Study	USA	519 NBA athletes	24.8 years (mean)	Not specified	Basketball	Professional athletes	Abnormal findings include T-wave inversions and prolonged QTc interval. T-wave inversion more common in athletes with higher left ventricular relative wall thickness	Not specified	The findings are specific to NBA athletes and may not be generalizable to athletes in other sports; limited serial follow-up data and a lack of comparative ECG data for nonathletes with similar biometrics to the NBA cohort.

ECG, electrocardiogram; LQTS, long QT syndrome; CSP, crista supraventricularis pattern; IRBBB, incomplete right bundle branch block; LVH, left ventricular hypertrophy; AHA, American Heart Association; PCVE, preparticipation cardiovascular evaluation; CVD, cardiovascular disease; TTE, transthoracic echocardiography; SCBC, screening for conditions at risk of sudden cardiac death; VA, ventricular arrhythmia; NBA, National Basketball Association.

Amongst the gender and age differences, there were several salient findings. Most studies showed males as having higher QRS voltages as well as longer QRS durations and higher PR intervals. In comparison, females had higher resting heart rates but longer QTc intervals. The incidence of abnormal ECG findings, however, was more often reported in males. Regarding age, adolescents who were regular in sports showed a lower prevalence of abnormal ECG findings compared to adult athletes. However, when the abnormalities were detected, they often manifested as increased QRS voltage or TWI. Additionally, adolescents engaged in high-intensity training beyond typical levels for their age group, showed cardiac changes such as right atrial enlargement, which were also indicated by an increase in P-wave duration (Table 2).

When considering sport-specific adaptations, athletes involved in endurance sports, such as marathon running and cycling, displayed a higher prevalence of sinus bradycardia, early repolarization, and right atrial enlargement. Strength athletes, such as those who lifted weights, were more likely to exhibit signs of LVH and increased QRS voltage. Athletes participating in mixed sports like soccer or basketball, displayed a combination of endurance and strength-related ECG changes, including both increased QRS voltage and sinus bradycardia (Table 2).

One of the most clinically significant findings, was that detraining effectively reversed certain ECG changes, such as prolonged QT interval and T-wave abnormalities. In some cases, these changes returned to baseline upon retraining. Moreover, a study reported a high false-positive rate when using standard ECG criteria to screen patients [19]. Notably, we found only one study that identified new ECG changes in athletes after COVID-19 infection, which included T-wave inversions and ST-segment depression (Table 2).

The included studies highlighted that youth athletes frequently exhibited ECG abnormalities, such as TWI, bradycardia, and prolonged QT intervals, with increased incidence during the COVID-19 pandemic due to the risk of myocarditis. Furthermore, exercise-induced acquired long QT syndrome (LQTS) was also noted, with ECG abnormalities resolving after detraining but recurring with high-intensity training. These findings highlight the necessity of comprehensive cardiac screening and monitoring in young athletes.

Extensive diagnostic evaluations—including cardiac imaging, Holter monitoring, and genetic testing—proved essential in identifying complex arrhythmias and distinguishing between congenital and acquired cardiac conditions across all studies, not just youth athletes. Studies also acknowledged the financial burden of ECG-inclusive screening programs but emphasized their vital role in preventing sudden cardiac death [13, 33].

Overall, the results indicate that ECG alterations, such as left ventricular hypertrophy, QT prolongation, and other cardiac changes, are frequently observed in athletes. These alterations generally revert to baseline after periods of detraining, while youth athletes exhibit vulnerability to COVID-19-related myocarditis and training-induced cardiac adaptations.

### 2.3 Discussion

This review was only partially successful in addressing all research questions. While the influence of specific sports on ECG changes and the impact of age differences were evident, only one study identified a particular risk factor unrelated to the type of sport. Nevertheless, the findings presented in this review provide valuable insights into the subject matter.

Accurately distinguishing between physiological adaptations and pathological conditions when interpreting ECG findings in athletes is paramount. Athletes frequently exhibit ECG changes as a direct consequence of cardiac adaptations to the increased physiological demands imposed by intense physical training. Common adaptations such as increased QRS voltage, sinus bradycardia, and early repolarization patterns are typically benign and reflect normal cardiac remodeling. However, more pronounced changes, particularly in athletes engaged in endurance or strength-based sports, such as LVH, present a greater challenge. LVH, characterized by elevated QRS amplitude and TWI, results from the heart’s adaptive response to meet the heightened oxygen demands of skeletal muscles by increasing left ventricular dimensions to maintain adequate cardiac output [6, 7].

The necessity of this differentiation extends beyond clinical relevance to financial implications. Misinterpreting physiological adaptations as pathological abnormalities can lead to significant financial burdens on healthcare systems, sports organizations, and athletes. Avoiding unwarranted diagnostic evaluations is essential to prevent escalating costs while ensuring that genuine pathological conditions are identified and managed appropriately. Therefore, accurate interpretation of ECG findings is critical not only for safeguarding athlete health but also for minimizing the economic strain associated with excessive medical examinations. Previous cost analyses of athlete ECG screening have not adequately considered various policies and practices across different countries and federations. Until all stakeholders convene to establish a unified approach that prioritizes athlete well-being, it remains challenging to draw definitive conclusions on the cost-effectiveness of widespread ECG screening. While it is impossible to assign a price to an athlete’s life, implementing a sport-specific risk stratification system, potentially integrated with predictive modeling, could enhance screening precision, reduce unnecessary tests, and help forecast the future risk of SCD through simulation-based technologies.

The distinction between HCM and arrhythmogenic right ventricular cardiomyopathy (ARVC) remains one of the most challenging aspects of ECG interpretation in athletes when TWI is present in the anterior leads. While these findings may indicate underlying cardiomyopathy, they can also represent benign physiological adaptations to intensive training. Similarly, prolonged QT intervals may be a hallmark of LQTS, a potentially life-threatening condition. Accurate differentiation is imperative, as misclassification can lead to unnecessary investigations or missed diagnoses that increase the risk of SCD [9, 10, 11]. The reversibility of many ECG changes following detraining as documented in several studies, supports the notion that numerous adaptations are physiological rather than pathological. Nevertheless, clinicians must remain vigilant, particularly when faced with deep TWI in multiple leads or markedly prolonged QT intervals, as these findings warrant further investigation to exclude underlying pathology [17, 34].

In this context, recent guidelines from the Italian Society of Sports Cardiology (SICSPORT) [35] offer essential insights into the interpretation of TWI in athletes. Recognizing the diagnostic challenge posed by TWI, these guidelines provide a framework for distinguishing between benign adaptations and serious cardiac pathologies, emphasizing the importance of TWI localization, clinical features, and demographic factors. The document highlights the need for comprehensive assessments and regular follow-ups, ensuring athlete safety while avoiding unnecessary restrictions and minimizing financial burdens associated with over-examination. African and Afro-Caribbean athletes show a higher prevalence of repolarization anomalies, such as TWI, especially in the anterior and inferior leads. Notably, black athletes are 2.5 times more likely to present with ECG abnormalities compared to white athletes, which are often misinterpreted as pathological. This increased prevalence of TWI in black athletes is typically a normal ethnic variant linked to the athlete’s heart but can lead to unnecessary testing and interventions if not correctly identified. A more nuanced approach to ECG interpretation in these populations is critical to prevent over-investigation and reduce the associated clinical and financial burden [36].

The cardiovascular adaptations induced by different sports reflect the unique hemodynamic and physiological demands on the athlete’s heart. This is particularly evident in endurance sports, where athletes such as marathon runners, cyclists, and swimmers experience significant volume overload due to prolonged aerobic activity. Consequently, the heart experiences structural remodeling, characterized by an increase in left ventricular cavity and wall thickness to enhance cardiac output and efficiency during sustained exercise. Endurance athletes commonly exhibit sinus bradycardia and early repolarization patterns on ECG, findings that were consistently reported across multiple studies [7, 8].

In contrast, strength-based sports such as weightlifting impose a predominant pressure overload on the cardiovascular system due to short but intense bursts of isometric activity. However, recent evidence suggests that even elite weightlifters typically do not develop significant myocardial hypertrophy under physiological training conditions. Echocardiographic data from Olympic athletes revealed that all weightlifters demonstrated normal left ventricular (LV) geometry, without signs of concentric or eccentric remodeling, despite high training volumes [37]. According to the 2023 Italian Cardiological Guidelines (COCIS) cardiology protocols for pre-participation evaluation, the diagnostic threshold for HCM is an LV wall thickness ≥15 mm, while the “grey zone” (13–15 mm) occurs in fewer than 3–5% of Caucasian male athletes but may be present in up to 18–20% of athletes of African descent. For Caucasian females, LV wall thickness typically does not exceed 12 mm, while in African females, the threshold is 13 mm. When wall thickness measurements exceed these physiological ranges, clinicians must consider the possibility of pathological hypertrophy (e.g., HCM) or non-physiological influences such as anabolic-androgenic steroid use [9, 36, 38]. Chronic use of anabolic substances has been linked to disproportionate increases in LV mass, impaired diastolic function, and the development of a proarrhythmic myocardial substrate, thereby mimicking or amplifying features of HCM [39]. Therefore, any finding of increased LV wall thickness in strength athletes warrants a comprehensive differential diagnosis that integrates ethnicity, training load, hypertrophy pattern, reversibility upon detraining, and a comprehensive assessment of potential doping history [40, 41].

Mixed sports exhibit a combination of cardiovascular adaptations associated with endurance and strength training, though to a lesser extent than specialized sports. For instance, football players may not sustain prolonged exertion comparable to marathon runners nor generate the intense pressure loads seen in weightlifters. Nonetheless, ECG findings in football players frequently demonstrate increased QRS voltage, indicative of strength-related adaptations, alongside sinus bradycardia, reflecting endurance training effects [4].

The classification of sports disciplines based on acute physiological responses and long-term cardiac remodeling further underscores the variability in cardiovascular adaptations across different athletic activities, as highlighted by the European Association of Preventive Cardiology (EAPC) [42]. Mixed sports, such as football, rugby, and basketball, are characterized by alternating phases of dynamic and static exertion, resulting in moderate cardiac remodeling marked by an increase in left ventricular cavity size with modest changes in wall thickness. This aligns with the observed ECG findings of increased QRS voltage and sinus bradycardia, reflecting the combined influence of endurance and strength demands.

The sport-specific nature of ECG changes necessitates interpretation criteria that account for the physiological demands of different sports. The latest International Criteria for Electrocardiographic Interpretation in Athletes [11] provides a robust framework for distinguishing physiological adaptations from pathological findings, though further refinement is required to accommodate the unique cardiovascular responses observed across diverse sports. Additionally, the influence of factors such as ethnicity and gender on ECG patterns warrants further investigation to enhance the accuracy and reliability of athlete screening.

Several studies have explored the impact of gender differences on ECG findings in athletes. It is well established that male athletes typically have greater muscle mass, both in skeletal muscles and in the myocardium. This increased myocardial mass contributes to enhanced electrical activity on ECG, as evidenced by higher QRS voltages and longer QRS durations observed in male athletes, a finding consistently reported in various studies [15, 23]. Furthermore, the greater overall muscle mass in male athletes increases circulatory demands during intense physical training, prompting further cardiac adaptations. In contrast, female athletes demonstrate higher resting heart rates and prolonged QTc intervals, which cannot be solely attributed to smaller myocardial mass. Hormonal factors, particularly the effects of estrogen on cardiac repolarization, alongside differences in autonomic tone—specifically lower vagal tone in females—also contribute to these gender-specific ECG variations.

The findings of this review highlight the unique challenges associated with cardiac screening in youth athletes. ECG abnormalities such as T-wave inversions, bradycardia, and prolonged QT intervals, were frequently observed, particularly during the COVID-19 pandemic, where potential myocarditis ECG changes were noted due to post-COVID syndrome. Although these changes are less prevalent in athletes, abnormalities such as TWI and ST-segment changes have been documented, indicating possible myocardial inflammation, especially myocarditis, which, while rare, presents an arrhythmogenic risk. To ensure proper diagnosis and mitigate potential risks, including SCD, ECG findings should be followed by more specific tests like cardiac magnetic resonance (CMR) [43]. The reversible nature of exercise-induced acquired LQTS, which resolves after detraining but recurs with high-intensity training, highlights the critical need for comprehensive cardiac monitoring in this population.

Pediatric athletes present challenges due to ongoing growth and maturation influencing cardiac adaptation. Ragazzoni *et al*. [12] emphasize the need for tailored ECG interpretation in young athletes and propose an algorithm to differentiate physiological adaptations from pathology, stressing age-specific guidelines for early detection of conditions like myocarditis and channelopathies to prevent SCD.

The review emphasizes the importance of balancing early detection of life-threatening conditions against the risk of overdiagnosis and unnecessary exclusion from sports. This is a call for further research and tailored recommendations for pediatric athletes.

The primary challenge in athlete screening lies in balancing the accurate detection of cardiac abnormalities with the financial and logistical burdens of widespread ECG use. The high false-positive rate often leads to unnecessary investigations, increased healthcare costs, and psychological stress for athletes. The European Society of Cardiology (ESC) and the Italian Society of Sports Cardiology have made significant contributions to refining ECG interpretation guidelines for athletes, promoting evidence-based standards to enhance diagnostic accuracy. Their efforts aim to address sport-specific, gender-specific, and age-related ECG variations, ensuring more reliable assessments and reducing false-positive rates. The EFSMA has also advocated for a standardized PPE protocol across Europe, including mandatory 12-lead ECGs, to provide equitable and comprehensive cardiac screening for all athletes. Notably, recreational athletes often train at intensities comparable to professionals, further underscoring the need for uniform screening standards. While comprehensive screening is often accessible to elite athletes through well-funded organizations, ensuring affordable PPE for all individuals participating in regular sports is essential. The financial burden of ECG screening can be prohibitive [13], especially in resource-limited settings, necessitating cost-effective solutions and specialized training for healthcare providers [34].

### 2.4 Limitations

This review offers valuable insights into ECG screening in athletes; however, several limitations must be acknowledged. The literature search was restricted to English-language studies published in the past decade, potentially excluding significant findings from non-English sources, particularly those from Eastern countries. The inclusion of only observational studies, due to a lack of randomized controlled trials, limits the strength of the evidence presented. Case reports were also excluded, as many involved athletes with pre-existing or newly developed cardiac conditions, which conflicted with the review’s inclusion criteria. Statistical analysis was hindered by the heterogeneity of study outcomes and methodologies, with some studies focusing on elite athletes and others on amateurs. Notably, the included studies varied widely in population demographics (age, sex, ethnicity), athletic level (elite vs. amateur), and sport type (e.g., endurance vs. strength). Furthermore, ECG interpretation criteria were not uniform—some studies employed the Seattle criteria or the 2017 International Recommendations—introducing variability in the classification and reporting of ECG abnormalities. Differences in study design (cross-sectional vs. longitudinal) and inconsistent follow-up durations further limit the comparability of findings and the ability to conclude long-term outcomes. Despite these limitations, the risk of bias was generally low across the included studies, although some were constrained by small sample sizes (Table 3, Ref. [13, 15, 16, 17, 18, 19, 20, 21, 22, 23, 24, 25, 26, 27, 28, 29, 30, 31, 32]).

**Table 3.** **Assessment of studies via Newcastle-Ottawa scale**.

Study	Selection (max 4 stars)	Comparability (max 2 stars)	Outcome/exposure (max 3 stars)	Total score (max 9 stars)
Albiński *et al*., 2022 [13]	★★★★	★★	★★★	9
Bessem *et al*., 2017 [15]	★★★★	★★	★★★	9
Bhatia *et al*., 2023 [16]	★★★★	★★	★★★	9
Dagradi *et al*., 2020 [17]	★★★★	★★	★★★	9
Diaz-Gonzalez *et al*., 2020 [18]	★★★★	★★	★★★	9
Dunn *et al*., 2015 [19]	★★★★	★★	★★★	9
Grimsmo *et al*., 2024 [20]	★★★	★★	★★	7
Herrera *et al*., 2022 [21]	★★★★	★★	★★★	9
Hupin *et al*., 2024 [22]	★★★★	★★	★★★	9
Idiazabal-Ayesa *et al*., 2023 [23]	★★★★	★★	★★★	9
Kaleta *et al*., 2018 [24]	★★★	★★	★★	7
Klein *et al*., 2023 [25]	★★★★	★★	★★★	9
Lasocka *et al*., 2021 [26]	★★★	★★	★★	7
McKinney *et al*., 2017 [27]	★★★★	★★	★★★	9
Narducci *et al*., 2020 [28]	★★★	★★	★★	7
Pentikäinen *et al*., 2022 [29]	★★★★	★★	★★★	9
Roberts *et al*., 2017 [30]	★★★	★★	★★	7
Šarčević and Tepavčević, 2021 [31]	★★★★	★★	★★★	9
Waase *et al*., 2018 [32]	★★★★	★★	★★★	9

Stars (★) represent the quality score assigned to each domain based on the Newcastle–Ottawa Scale (NOS). A maximum of 4 stars can be awarded for selection, 2 for comparability, and 3 for outcome/exposure, totaling up to 9 stars. Higher scores indicate better methodological quality.

## 3. Conclusions

The interpretation of ECG changes in athletes presents a complex challenge, demanding clear differentiation between physiological adaptations and pathological abnormalities. This systematic review highlights sport-specific, gender-based age-related ECG variations and persistent high false-positive rates during screenings. The recent EC Delegated Act (April 2024) recognizing SEM as a primary specialty under European Union (EU) law reinforces the need for comprehensive training of SEM physicians. Long-term, multicentric studies across diverse populations, including pediatric and veteran athletes, are essential to refining ECG interpretation standards. Integrating artificial intelligence (AI) technology can enhance accuracy, reduce costs, and identify subtle cardiac risks, making large-scale screenings more efficient and affordable. Future research could benefit from expanding the search to include multilingual sources, particularly non-English language publications, to further enhance the universality and global applicability of the findings. A refined risk stratification model based on sports discipline, along with a comprehensive framework for follow-up, is essential for improving ECG screening accuracy and ensuring proper cardiovascular management in athletes. Collaborative efforts between clinicians, researchers, and policymakers are vital for developing sport-specific, cost-effective ECG guidelines. Equipping both current and future SEM physicians with advanced diagnostic tools and AI-driven solutions will improve athlete care without imposing additional costs, supporting accessible and standardized cardiac evaluations across all sports disciplines. This forward-thinking approach will drive innovation in sports cardiology, advance athlete health management, and ensure that physicians are well-prepared to meet the evolving challenges of sports cardiology.

## Availability of Data and Materials

Data sharing is not applicable as no data were generated or analyzed.

## Supplementary Material

Supplementary material associated with this article can be found, in the online version, at https://doi.org/10.31083/RCM38209.

## Appendix

See Table 4.

**Table 4.** **Search strategy outcomes on PubMed**.

Search number	Query	Sort By	Filters	Search Details	Results	Time
1	(electrocardiography [MeSH Terms]) AND (athletes [MeSH Terms]) AND (y_10 [Filter])	First author	In the last 10 years, observational study	(“electrocardiography” [MeSH Terms] AND “athletes” [MeSH Terms] AND “2015/02/10 00:00”:“3000/01/01 05:00” [Date - Publication]) AND ((y_10 [Filter]) AND (observationalstudy [Filter]))	24	6:34:51
2	(electrocardiography [MeSH Terms]) AND (“sports” [MeSH Terms])	First author	In the last 10 years, observational study	(“electrocardiography” [MeSH Terms] AND “sports” [MeSH Terms]) AND ((y_10 [Filter]) AND (observationalstudy [Filter]))	28	6:35:35
3	(((electrocardiography [MeSH Terms]) AND (strength sports [Title/Abstract])) OR (endurance sports [Title/Abstract])) OR (mixed sports [Title/Abstract])	First author	In the last 10 years, observational study	((“electrocardiography” [MeSH Terms] AND “strength sports” [Title/Abstract]) OR “endurance sports” [Title/Abstract] OR “mixed sports” [Title/Abstract]) AND ((y_10 [Filter]) AND (observationalstudy [Filter]))	10	6:36:59
4	((electrocardiography [MeSH Terms]) AND (athletes [MeSH Terms]) AND (y_10 [Filter]) AND ((y_10 [Filter]) AND (observationalstudy [Filter]))) AND (ECG changes [Title/Abstract])	First author	In the last 10 years, observational study	(“electrocardiography” [MeSH Terms] AND “athletes” [MeSH Terms] AND “2015/02/10 00:00”:“3000/01/01 05:00” [Date - Publication] AND (“2015/02/10 00:00”:“3000/01/01 05:00” [Date - Publication] AND “observational study” [Publication Type]) AND “ecg changes” [Title/Abstract]) AND ((y_10 [Filter]) AND (observationalstudy [Filter]))	3	6:38:56
5	((electrocardiography [MeSH Terms]) AND (athletes [MeSH Terms]) AND (y_10 [Filter]) AND ((y_10 [Filter]) AND (observationalstudy [Filter]))) AND (screening [Title/Abstract])	First author	In the last 10 years, observational study	(“electrocardiography” [MeSH Terms] AND “athletes” [MeSH Terms] AND “2015/02/10 00:00”:“3000/01/01 05:00” [Date - Publication] AND (“2015/02/10 00:00”:“3000/01/01 05:00” [Date - Publication] AND “observational study” [Publication Type]) AND “screening” [Title/Abstract]) AND ((y_10 [Filter]) AND (observationalstudy [Filter]))	14	6:39:31

MeSH, Medical Subject Headings.

## References

[1] Martis RJ, Acharya UR, Adeli H (2014). Current methods in electrocardiogram characterization. *Computers in Biology and Medicine*.

[2] Ionescu AM, Pitsiladis YP, Rozenstoka S, Bigard X, Löllgen H, Bachl N (2021). Preparticipation medical evaluation for elite athletes: EFSMA recommendations on standardised preparticipation evaluation form in European countries. *BMJ Open Sport & Exercise Medicine*.

[3] Uberoi A, Stein R, Perez MV, Freeman J, Wheeler M, Dewey F (2011). Interpretation of the electrocardiogram of young athletes. *Circulation*.

[4] Drezner JA, Asif IM, Owens DS, Prutkin JM, Salerno JC, Fean R (2012). Accuracy of ECG interpretation in competitive athletes: the impact of using standised ECG criteria. *British Journal of Sports Medicine*.

[5] Smaranda AM, Drăgoiu TS, Caramoci A, Afetelor AA, Ionescu AM, Bădărău IA (2024). Artificial Intelligence in Sports Medicine: Reshaping Electrocardiogram Analysis for Athlete Safety-A Narrative Review. *Sports (Basel, Switzerland)*.

[6] Coote JH, White MJ (2015). CrossTalk proposal: bradycardia in the trained athlete is attributable to high vagal tone. *The Journal of Physiology*.

[7] Pelliccia A, Maron MS, Maron BJ (2012). Assessment of left ventricular hypertrophy in a trained athlete: differential diagnosis of physiologic athlete’s heart from pathologic hypertrophy. *Progress in Cardiovascular Diseases*.

[8] Lovic D, Narayan P, Pittaras A, Faselis C, Doumas M, Kokkinos P (2017). Left ventricular hypertrophy in athletes and hypertensive patients. *Journal of Clinical Hypertension (Greenwich, Conn.)*.

[9] Malhotra A, Sharma S (2017). Hypertrophic Cardiomyopathy in Athletes. *European Cardiology*.

[10] Drezner JA, Ackerman MJ, Anderson J, Ashley E, Asplund CA, Baggish AL (2013). Electrocardiographic interpretation in athletes: the ‘Seattle criteria’. *British Journal of Sports Medicine*.

[11] Drezner JA, Sharma S, Baggish A, Papadakis M, Wilson MG, Prutkin JM (2017). International criteria for electrocardiographic interpretation in athletes: Consensus statement. *British Journal of Sports Medicine*.

[12] Ragazzoni GL, Cavigli L, Cavarretta E, Maffei S, Mandoli GE, Pastore MC (2023). How to evaluate resting ECG and imaging in children practising sport: a critical review and proposal of an algorithm for ECG interpretation. *European Journal of Preventive Cardiology*.

[13] Albiński M, Saubade M, Menafoglio A, Meyer P, Capelli B, Perrin T (2022). Diagnostic yield and cost analysis of electrocardiographic screening in Swiss paediatric athletes. *Journal of Science and Medicine in Sport*.

[14] Albiński M, Saubade M, Benaim C, Menafoglio A, Meyer P, Capelli B (2021). Impact of early sports specialisation on paediatric ECG. *Scandinavian Journal of Medicine & Science in Sports*.

[15] Bessem B, de Bruijn MC, Nieuwland W (2017). Gender differences in the electrocardiogram screening of athletes. *Journal of Science and Medicine in Sport*.

[16] Bhatia RT, Malhotra A, MacLachlan H, Gati S, Marwaha S, Chatrath N (2023). Prevalence and diagnostic significance of de-novo 12-lead ECG changes after COVID-19 infection in elite soccer players. *Heart (British Cardiac Society)*.

[17] Dagradi F, Spazzolini C, Castelletti S, Pedrazzini M, Kotta MC, Crotti L (2020). Exercise Training-Induced Repolarization Abnormalities Masquerading as Congenital Long QT Syndrome. *Circulation*.

[18] Diaz-Gonzalez L, Bruña V, Velásquez-Rodriguez J, Valenzuela PL, Valero-Masa MJ, González-Saldívar H (2020). Young athletes’ ECG: Incomplete right bundle branch block vs crista supraventricularis pattern. *Scandinavian Journal of Medicine & Science in Sports*.

[19] Dunn TP, Pickham D, Aggarwal S, Saini D, Kumar N, Wheeler MT (2015). Limitations of Current AHA Guidelines and Proposal of New Guidelines for the Preparticipation Examination of Athletes. *Clinical Journal of Sport Medicine: Official Journal of the Canadian Academy of Sport Medicine*.

[20] Grimsmo J, Haugaa KH, Popovic I, Lie ØH, Solberg EE (2024). Value of preparticipation cardiovascular evaluation of master athletes by self-reported symptoms and cardiovascular risk-score. *Scandinavian Cardiovascular Journal: SCJ*.

[21] Herrera C, Bruña V, Comella A, de la Rosa A, Díaz-González L, Ruiz-Ortiz M (2022). Left atrial enlargement in competitive athletes and atrial electrophysiology. *Revista Espanola De Cardiologia (English Ed.)*.

[22] Hupin D, Oriol M, Laukkanen JA, Abraham P, Dulac N, Laugier S (2024). Screening Sportsmen and Sportswomen Over Age 35: The Relevance of an Exercise Electrocardiogram. Data From the SEEPRED Study. *Scandinavian Journal of Medicine & Science in Sports*.

[23] Idiazabal-Ayesa U, Ramírez-Vélez R, Sanz-de la Garza M, Izquierdo M (2023). Electrocardiographic findings in pediatric versus young-adolescent athletes: A comparative analysis using general international criteria. *International Journal of Cardiology*.

[24] Kaleta AM, Lewicka E, Dąbrowska-Kugacka A, Lewicka-Potocka Z, Wabich E, Szerszyńska A (2018). Electrocardiographic abnormalities in amateur male marathon runners. *Advances in Clinical and Experimental Medicine: Official Organ Wroclaw Medical University*.

[25] Klein CF, Petek BJ, Moulson N, Baggish AL, Churchill TW, Harmon KG (2023). Non-COVID-19 cardiovascular pathology from return-to-play screening in college athletes after COVID-19. *Heart (British Cardiac Society)*.

[26] Lasocka Z, Dąbrowska-Kugacka A, Kaleta AM, Lewicka-Potocka Z, Faran A, Szołkiewicz E (2021). Electrocardiographic Changes in Male and Female Amateur Marathon Runners: A Comparison Study. *International Journal of Sports Medicine*.

[27] McKinney J, Lithwick DJ, Morrison BN, Nazzari H, Luong M, Fordyce CB (2017). Detecting Underlying Cardiovascular Disease in Young Competitive Athletes. *The Canadian Journal of Cardiology*.

[28] Narducci ML, Pelargonio G, La Rosa G, Inzani F, d’Amati G, Novelli V (2020). Role of extensive diagnostic workup in young athletes and nonathletes with complex ventricular arrhythmias. *Heart Rhythm*.

[29] Pentikäinen H, Toivo K, Kokko S, Alanko L, Heinonen OJ, Nylander T (2022). Resting electrocardiogram and blood pressure in young athletes and nonathletes: A 4-year follow-up. *Clinical Physiology and Functional Imaging*.

[30] Roberts WO, Schwartz RS, Kraus SM, Schwartz JG, Peichel G, Garberich RF (2017). Long-Term Marathon Running Is Associated with Low Coronary Plaque Formation in Women. *Medicine and Science in Sports and Exercise*.

[31] Šarčević Z, Tepavčević A (2021). Increased number of electrocardiogram findings requiring additional cardiac examination in young athletes during the coronavirus disease 2019 pandemic: a case series. *The Journal of International Medical Research*.

[32] Waase MP, Mutharasan RK, Whang W, DiTullio MR, DiFiori JP, Callahan L (2018). Electrocardiographic Findings in National Basketball Association Athletes. *JAMA Cardiology*.

[33] Smaranda AM, Caramoci A, Drăgoiu TS, Bădărău IA (2025). ECG Evolution in Elite Gymnasts: A Retrospective Analysis of Training Adaptations, Risk Prediction, and PPE Optimization. *Diagnostics*.

[34] Rakhmanov Y, Toktarbay B, Khamitova Z, Salustri A (2024). ECG in Athletes. Technology in Sports - Recent Advances, New Perspectives and Application. *IntechOpen*.

[35] Palermi S, Tardini L, Graziano F, Bianco M, Bina A, Castelletti S (2025). Interpretation and management of T wave inversion in athletes: An expert opinion statement of the Italian Society of Sports Cardiology (SICSPORT). *International Journal of Cardiology*.

[36] Ozo U, Sharma S (2020). The Impact of Ethnicity on Cardiac Adaptation. *European Cardiology*.

[37] Di Gioia G, Ferrera A, Maestrini V, Monosilio S, Squeo MR, Lemme E (2024). Cardiac Adaptation in Power Athletes: Differential Impact of Judo and Weightlifting. *Journal of Clinical Medicine*.

[38] Maxwell JD, Oxborough D (2025). The athletes heart-from acute stimulus to chronic adaptation. *British Medical Bulletin*.

[39] Adami PE, Koutlianos N, Baggish A, Bermon S, Cavarretta E, Deligiannis A (2022). Cardiovascular effects of doping substances, commonly prescribed medications and ergogenic aids in relation to sports: a position statement of the sport cardiology and exercise nucleus of the European Association of Preventive Cardiology. *European Journal of Preventive Cardiology*.

[40] Pittaras A, Faselis C, Doumas M, Grassos C, Kokkinos P (2023). Physical Activity and Cardiac Morphologic Adaptations. *Reviews in Cardiovascular Medicine*.

[41] Sharma S, Chandra N, Papadakis M Cardiac adaptations to intense physical exercise in African/Afro-Carribean athletes. https://www.escardio.org/Journals/E-Journal-of-Cardiology-Practice/Volume-8/Cardiac-adaptations-to-intense-physical-exercise-in-African-Afro-Carribean-athle.

[42] Pelliccia A, Caselli S, Sharma S, Basso C, Bax JJ, Corrado D (2018). European Association of Preventive Cardiology (EAPC) and European Association of Cardiovascular Imaging (EACVI) joint position statement: recommendations for the indication and interpretation of cardiovascular imaging in the evaluation of the athlete’s heart. *European Heart Journal*.

[43] Laranjeira TA, Menezes AS (2022). A Systematic Review of Post-COVID Electrocardiographic Changes in Young Athletes. *Cureus*.

