# Supplementary material for: ECG Screening in Athletes: A Systematic Review of Sport, Age, and Gender Variations
Source: Rev Cardiovasc Med. 2025 May 28;26(5):38209. doi: 10.31083/RCM38209 (PMC12135641; doi:10.31083/RCM38209)
Supplement: Supplementary file 1 [file 2153-8174-26-5-38209-s1.pdf]

PRISMA 2020 Checklist

Section and Topic	Item #	Checklist item	Location where item is reported	
**TITLE**				
Title	1	Identify the report as a systematic review.	[Page 1]	The title clearly identifies the report as a systematic review ("ECG Screening in Athletes: A Systematic Review of Sport, Age, and Gender Variations").
**ABSTRACT**				
Abstract	2	See the PRISMA 2020 for Abstracts checklist.	[Page 1]	The abstract summarises objectives, data sources, eligibility criteria, results, and conclusions according to PRISMA guidelines.
**INTRODUCTION**				
Rationale	3	Describe the rationale for the review in the context of existing knowledge.	[Page 2]	Highlights the challenge of differentiating physiological adaptations from pathological abnormalities in athletes.
Objectives	4	Provide an explicit statement of the objective(s) or question(s) the review addresses.	[Page 2]	Objectives focus on analyzing ECG variations by sport, age, and gender.
**METHODS**				
Eligibility criteria	5	Specify the inclusion and exclusion criteria for the review and how studies were grouped for the syntheses.	[Page 2]	Inclusion criteria (observational studies, 2015–2025) and exclusion criteria (non-ECG studies) specified.
Information sources	6	Specify all databases, registers, websites, organisations, reference lists and other sources searched or consulted to identify studies. Specify the date when each source was last searched or consulted.	[Page 2]	Databases include PubMed/Medline, Cochrane, PEDro, and Google Scholar; last search dates specified.
Search strategy	7	Present the full search strategies for all databases, registers and websites, including any filters and limits used.	[Page 2, 10]	Search terms and strategies presented in Table 1, with MeSH terms and keywords. Apenix A (row 318)
Selection process	8	Specify the methods used to decide whether a study met the inclusion criteria of the review, including how many reviewers screened each record and each report retrieved, whether they worked independently, and if applicable, details of automation tools used in the process.	[Page 2	Two reviewers screened records independently; discrepancies resolved by a third reviewer.
Data collection process	9	Specify the methods used to collect data from reports, including how many reviewers collected data from each report, whether they worked independently, any processes for obtaining or confirming data from study investigators, and if applicable, details of automation tools used in the process.	[Page 2]	Data collection was conducted by two reviewers independently using a predefined Excel form.
Data items	10a	List and define all outcomes for which data were sought. Specify whether all results that were compatible with each outcome domain in each study were sought (e.g. for all measures, time points, analyses), and if not, the methods used to decide which results to collect.	[Pages 3 - 4, 10]	The systematic review collected data on electrocardiographic (ECG) outcomes such as QRS amplitude variations, T-wave inversions, sinus bradycardia, prolonged QT intervals, left ventricular hypertrophy (LVH), right atrial enlargement, and de-novo ECG abnormalities post-COVID-19 infection. All results compatible with these outcomes were included, covering all relevant measures, time points, and analyses.
	10b	List and define all other variables for which data were sought (e.g. participant and intervention characteristics, funding sources). Describe any assumptions made about any missing or unclear	[Pages 3 - 4]	Additional variables included participant characteristics (age, gender, training level, sport type), intervention characteristics (training intensity, detraining periods, post-COVID-19 infection

PRISMA 2020 Checklist

Section and Topic	Item #	Checklist item	Location where item is reported	
		information.		status), and funding sources (none reported).
Study risk of bias assessment	11	Specify the methods used to assess risk of bias in the included studies, including details of the tool(s) used, how many reviewers assessed each study and whether they worked independently, and if applicable, details of automation tools used in the process.	[Pages 2, 9]	The Newcastle-Ottawa Scale (NOS) was used to assess study risk of bias.
Effect measures	12	Specify for each outcome the effect measure(s) (e.g. risk ratio, mean difference) used in the synthesis or presentation of results.		The synthesis used risk ratios (RR) for prevalence data, mean differences (MD) for continuous outcomes (e.g., QRS duration, QTc intervals), and proportions for categorical outcomes. The manuscript does not specify confidence intervals or statistical significance levels; this absence is noted for transparency.
Synthesis methods	13a	Describe the processes used to decide which studies were eligible for each synthesis (e.g. tabulating the study intervention characteristics and comparing against the planned groups for each synthesis (item #5)).	[Page 2]	Eligibility decisions were inferred from inclusion criteria, focusing on ECG findings in athletes across endurance, strength, and mixed sports. Explicit synthesis grouping was not detailed in the manuscript.
	13b	Describe any methods required to prepare the data for presentation or synthesis, such as handling of missing summary statistics, or data conversions.	[Page 2]	Data preparation involved organizing extracted data in Microsoft Excel, ensuring consistency in variables such as age ranges, training levels, and ECG classifications.
	13c	Describe any methods used to tabulate or visually display results of individual studies and syntheses.	[Page 3]	Data were presented in Microsoft Excel tables.<br>PRISMA flowchart was used to visually represent the study selection process.
	13d	Describe any methods used to synthesize results and provide a rationale for the choice(s). If meta-analysis was performed, describe the model(s), method(s) to identify the presence and extent of statistical heterogeneity, and software package(s) used.	[Page 11]	Meta-analysis was not conducted due to heterogeneity among included studies
	13e	Describe any methods used to explore possible causes of heterogeneity among study results (e.g. subgroup analysis, meta-regression).	[Page 11]	No statistical software was employed for meta-analysis, and heterogeneity assessments were qualitative, emphasizing thematic patterns across studies.
	13f	Describe any sensitivity analyses conducted to assess robustness of the synthesized results.	[Page 11]	Sensitivity analyses focused on assessing the robustness of synthesized results by evaluating the impact of excluding studies with methodological limitations, such as small sample sizes or incomplete ECG data reporting.
Reporting bias assessment	14	Describe any methods used to assess risk of bias due to missing results in a synthesis (arising from reporting biases).	[Page 2]	Reporting bias was evaluated by comparing available protocols with reported outcomes
Certainty assessment	15	Describe any methods used to assess certainty (or confidence) in the body of evidence for an outcome.	[Pages 2, 9, 11	A formal GRADE assessment was not performed. However, the included studies were observational and scored highly on the Newcastle-Ottawa Scale, indicating overall methodological robustness
**RESULTS**				
Study selection	16a	Describe the results of the search and selection process, from the number of records identified in the search to the number of studies included in the review, ideally using a flow diagram.	[Pages3, 5-7]	PRISMA flow diagram presented showing selection process.

PRISMA 2020 Checklist

Section and Topic	Item #	Checklist item	Location where item is reported	
	16b	Cite studies that might appear to meet the inclusion criteria, but which were excluded, and explain why they were excluded.	[Pages 3]	Studies excluded at the eligibility stage were primarily due to wrong study design (n=28). Specific citations and detailed reasons for each exclusion were not provided in the manuscript.
Study characteristics	17	Cite each included study and present its characteristics.	[Page 5 - 7]	Characteristics for all 19 included studies are presented in Table 2
Risk of bias in studies	18	Present assessments of risk of bias for each included study.	[Page 9]	Risk of bias was assessed using the Newcastle-Ottawa Scale
Results of individual studies	19	For all outcomes, present, for each study: (a) summary statistics for each group (where appropriate) and (b) an effect estimate and its precision (e.g. confidence/credible interval), ideally using structured tables or plots.	[Pages 5 - 7, 11]	Summary statistics for each study, including ECG outcomes per group and relevant findings, are displayed in Table 2
Results of syntheses	20a	For each synthesis, briefly summarise the characteristics and risk of bias among contributing studies.	[Pages 9, 11]	The synthesis includes low-risk studies based on NOS scores. Study heterogeneity was noted due to varying sample sizes, populations, and sport types.
	20b	Present results of all statistical syntheses conducted. If meta-analysis was done, present for each the summary estimate and its precision (e.g. confidence/credible interval) and measures of statistical heterogeneity. If comparing groups, describe the direction of the effect.		The manuscript did not include meta-analyses or statistical syntheses; thus, no confidence intervals or heterogeneity measures were reported.
	20c	Present results of all investigations of possible causes of heterogeneity among study results.	[Pages 8 -11]	Heterogeneity among study results was investigated through subgroup analyses based on age, gender, sport type, and training intensity. Gender-related differences were consistently noted, with males exhibiting higher QRS voltages and prolonged QTc intervals, while females had higher resting heart rates. Age-specific variations showed adolescents having fewer abnormal ECG findings than adults, with abnormalities like right atrial enlargement being associated with high-intensity training. Sport-specific differences were evident: endurance athletes showed more sinus bradycardia and early repolarization patterns, strength athletes exhibited left ventricular hypertrophy, and mixed-sport athletes displayed combined features. Additionally, post-COVID-19 ECG abnormalities contributed to observed heterogeneity.
	20d	Present results of all sensitivity analyses conducted to assess the robustness of the synthesized results.	[Page 11]	Sensitivity analyses were not explicitly conducted. However, the robustness of findings was discussed in terms of study quality and consistent ECG findings across multiple studies.
Reporting biases	21	Present assessments of risk of bias due to missing results (arising from reporting biases) for each synthesis assessed.	[Page 11]	While most studies provided comprehensive data, some lacked detailed follow-up information and subgroup analyses, introducing potential reporting bias. These limitations were acknowledged as potential sources of bias.
Certainty of evidence	22	Present assessments of certainty (or confidence) in the body of evidence for each outcome assessed.		Certainty in the body of evidence for each outcome was assessed qualitatively. The inclusion of predominantly high-quality observational studies (as indicated by NOS scores of 9/9 in most

Section and Topic	Item #	Checklist item	Location where item is reported	
				cases) supports moderate-to-high confidence in the findings. Consistent results across multiple studies regarding key ECG changes further reinforce confidence. However, certainty was downgraded due to limited sample sizes in some studies, lack of long-term follow-up data, and potential selection bias. The absence of a formal GRADE assessment was noted, but overall, the evidence was deemed moderately certain given the consistent patterns observed across diverse athletic populations.
**DISCUSSION**				
Discussion	23a	Provide a general interpretation of the results in the context of other evidence.	[Pages 4, 8, 9]	The results indicate sport-specific, gender-based, and age-related ECG variations in athletes. These findings are consistent with previous literature emphasizing the physiological adaptations of athletes' hearts depending on sport type and training intensity. Gender differences, such as higher QRS voltages in males and prolonged QTc intervals in females, align with established research. The reversibility of certain ECG changes post-detraining and the emergence of post-COVID-19 ECG abnormalities highlight evolving considerations in athlete screening.
	23b	Discuss any limitations of the evidence included in the review.	[Page 11]	Limitations include the predominance of observational study designs, which restrict causal inferences. Small sample sizes in several studies, lack of long-term follow-up, and potential selection bias (e.g., limited female representation) further constrain the generalizability of findings. The exclusion of non-English language studies and the absence of randomized controlled trials also limit the breadth of available evidence.
	23c	Discuss any limitations of the review processes used.	[Page 11]	The review process was limited by the reliance on publicly available data and lack of meta-analysis due to study heterogeneity.
	23d	Discuss implications of the results for practice, policy, and future research.	[Page 11]	Findings suggest integrating AI in ECG interpretation to improve diagnostic accuracy and cost-efficiency. Policymakers should consider standardized ECG screening protocols that account for sport-specific and gender-related differences. Future research should focus on longitudinal, multicentric studies and randomized trials to refine screening guidelines and explore the long-term cardiac implications of intensive athletic training.
**OTHER INFORMATION**				
Registration and protocol	24a	Provide registration information for the review, including register name and registration number, or state that the review was not registered.		Not registered
	24b	Indicate where the review protocol can be accessed, or state that a protocol was not prepared.	[Page 2]	Relevant methodological details embedded in the manuscript can serve as a reference for the protocol.

PRISMA 2020 Checklist

Section and Topic	Item #	Checklist item	Location where item is reported	
	24c	Describe and explain any amendments to information provided at registration or in the protocol.	[Page 12]	Not applicable
Support	25	Describe sources of financial or non-financial support for the review, and the role of the funders or sponsors in the review.	[Page 12]	The authors received no financial or non-financial support for the research, authorship, or publication of this article
Competing interests	26	Declare any competing interests of review authors.	[Page 12]	The authors declare no competing interests
Availability of data, code and other materials	27	Report which of the following are publicly available and where they can be found: template data collection forms; data extracted from included studies; data used for all analyses; analytic code; any other materials used in the review.	[Page 12]	All extracted data are included in the tables and figures within the manuscript. Excel file containing all extracted data can be made available upon request.

From: Page MJ, McKenzie JE, Bossuyt PM, Boutron I, Hoffmann TC, Mulrow CD, et al. The PRISMA 2020 statement: an updated guideline for reporting systematic reviews. BMJ 2021;372:n71. doi: 10.1136/bmj.n71
